# Right Lower Quadrant Abdominal Pain in a Patient with Prior Ventriculoperitoneal Shunting: Consider the Tip!

**DOI:** 10.1155/2012/253027

**Published:** 2012-02-20

**Authors:** Petros Charalampoudis

**Affiliations:** Second Propedeutic Department of Surgery, Laiko Hospital, Athens Medical School, 18 Agiou Thoma Street, 11527 Athens, Greece

## Abstract

*Introduction*. Ventriculoperitoneal (VP) shunting is the treatment of choice for nonobstructive hydrocephalus. In patients with such a device, right lower quadrant abdominal pain can puzzle the surgeon, posing a differential diagnostic problem among appendicitis, nonsurgical colicky pain, and primary shunt catheter tip infection. Treatment is different in either case. *Presentation of Case*. We hereby present a case of a young woman with prior ventriculoperitoneal shunt positioning who presented to our department with right lower quadrant abdominal pain. The patient underwent a 24-hour observation including a neurosurgery consult in order to exclude acute appendicitis and VP shunt tip infection. Twenty four hours later, the patient's symptomatology improved, and she was discharged with the diagnosis of atypical colicky abdominal pain seeking a gastroenterologist consult. *Discussion*. This case supports that when a patient with prior VP shunting presents with right lower quadrant abdominal pain, differential diagnosis can be tricky for the surgeon. *Conclusion*. Apart from acute appendicitis, primary or secondary VP catheter tip infection must be considered because the latter can be disastrous.

## 1. Introduction

The mainstay of nonobstructive hydrocephalus treatment is ventriculoperitoneal shunting, which derives the cerebrospinal fluid into the abdominal cavity. The tip of this catheter is usually placed in the right lower quadrant, and therefore, primary infection of the catheter can sometimes mimic acute appendicitis, while the true hazard in this case can be an ascending meningitis with mental status alterations alongside. Patients with a ventriculoperitoneal catheter can present though with a true appendicitis at any age. In such cases, the surgeon needs to clarify the true source of intra-abdominal infection/inflammation and decide accordingly what to do next. The dilemma “appendectomy or catheter revision” seeks an answer when this rare, special, and challenging patient category is admitted with right lower quadrant pain.

## 2. Presentation of Case

A 31-year-old woman was admitted to our Emergency Surgical Department with right lower quadrant abdominal pain and mild nausea. Her past medical history was marked by a ventriculoperitoneal shunt for nonobstructive hydrocephalus at the age of four. Personal medical history was otherwise free. Her temperature at the time of admission to the hospital was normal. She reported a normal appetite. Physical examination revealed a mild tenderness on the right lower quadrant, but rebound tenderness was absent. White blood cell count was 11,300 with a left shift of 81.5% neutrophils. Plain abdominal films showed clearly the trajectory of the VP shunt into the peritoneal cavity and the tip of the catheter in the right lower quadrant. Abdominal ultrasound showed a mild inflammation of the caecum. Due to possible catheter tip infection, we sought a complementary neurosurgery consult, which advised for watchful observation over the next 24 hours. Mental status was stable, and no sign of meningitis was present. The patient was discharged 24 hours later with a diagnosis of atypical colicky abdominal pain as her symptomatology improved and leukocytosis with left shift as resolved completely; acute appendicitis along with catheter tip infection was excluded.

## 3. Discussion

Hydrocephalus, with an overall age prevalence of 1.5% can have several congenital or acquired etiologies [1]. Positioning of a ventriculoperitoneal (VP) shunt to derive the cerebrospinal fluid into the abdominal cavity has brightened the outlook for children with nonobstructive hydrocephalus in the modern era. [2]. In cases of obstructive hydrocephalus, caused by aqueductal stenosis or space-occupying lesions, third ventriculostomy is a most effective treatment [3]. A long-time standing VP shunt intra-abdominally cannot run always uneventfully. Several shunt-related complications have been described, including *Staphylococcus epidermidis* infection with primary peritonitis, small bowel obstruction and formation of a cerebrospinal fluid pseudocyst in the shunt tip area [4, 5]. Infected VP shunts increase the risk of meningitis, shunt malfunction, and mental status changes [1]. Shunted patients may also present with any abdominal pathology unrelated to the shunt. Of note, the tip of the catheter frequently ends up in the right lower quadrant, but it could end up anywhere in the peritoneum since it is usually placed blindly. Nevertheless, as the tip of the VP catheter is often placed in the right lower quadrant, a primary infection of the shunt can mimic acute appendicitis, while a primarily pathologic appendix can puzzle the surgeon regarding the origin of the pain on a patient with prior history of VP shunt positioning. In other words, catheter infection with primary localized peritonitis—a shunt-related pathology—and true acute appendicitis—a shunt-unrelated pathology—poses a rare but crucial differential diagnostic problem regarding this category of patients and requires a skilled diagnostic workup paired with a competent clinical judgement [1, 6]. It has been shown that in case of true acute appendicitis in a patient with prior VP shunting, treatment of the primary cause of inflammation, that is, the appendix, must be carried out as in any other patient, that is, appendectomy either laparoscopically or by McBurney laparotomy. [1]. In most cases, the VP shunt is unaffected and left in place after careful perioperative inspection, while the appendix is removed [1, 6]. However, when primary shunt infection is the cause of peritonitis, the authors consent to either a conservative treatment with intravenous antibiotics or opt among removal, reposition (ventriculoatrial shunt conversion), and temporary shunt exteriorization [1, 6–8]. Routine appendectomy in these cases is unnecessary [2]. Ventriculoperitoneal shunt tapping is also considered in selected cases of shunt malfunction. The most sensitive indicators for shunt obstruction are lethargy and irritability, followed by headache and vomiting [9]. In our case, the patient did not have any of the aforementioned signs or symptoms, and therefore, the consultant neurosurgeon opted for a 24-hour watchful observation, which proved effective as the patient's clinical manifestations and neutrophil-shifted leukocytosis were resolved completely in 24 hours.

## 4. Conclusion

This rare and interesting case further supports that a patient with a history of prior ventriculoperitoneal can puzzle the surgeon when presenting with right lower quadrant abdominal pain. Infection of the catheter tip can prove detrimental and should always be considered in this rare group of patients. Unnecessary appendectomy must be avoided when the primary cause of peritonitis is the shunt catheter and not the appendix, while a multidisciplinary team including a neurosurgeon should be alert of the possible hazards of such infection, that is, ascending meningitis. Catheter revision, shunt tapping, or even temporary exteriorization should be considered in these cases.
